# Recombinant Silk Proteins with Additional Polyalanine Have Excellent Mechanical Properties

**DOI:** 10.3390/ijms22041513

**Published:** 2021-02-03

**Authors:** Shuo Zhao, Xiaogang Ye, Meiyu Wu, Jinghua Ruan, Xiaoxiao Wang, Xiaoli Tang, Boxiong Zhong

**Affiliations:** College of Animal Sciences, Zhejiang University, Hangzhou 310058, China; asd8899176@163.com (S.Z.); 11817001@zju.edu.cn (X.Y.); 11617008@zju.edu.cn (M.W.); jinghuaryan@icloud.com (J.R.); 21717412@zju.edu.cn (X.W.); xltang2017@163.com (X.T.)

**Keywords:** silkworm, spider, transgene, polyalanine, β-sheets, silk strength

## Abstract

This paper explores the structures of exogenous protein molecules that can effectively improve the mechanical properties of silkworm silk. Several transgenic vectors fused with the silkworm fibroin light chain and type 3 repeats in different multiples of the ampullate dragline silk protein 1 (MaSp1) from black widow spider with different lengths of the polyalanine motifs were constructed for this study. Transgenic silkworms were successfully obtained by *piggy*Bac-mediated microinjection. Molecular detection showed that foreign proteins were successfully secreted and contained within the cocoon shells. According to the prediction of PONDR^®^ VSL2 and PONDR^®^ VL-XT, the type 3 repeats and the polyalanine motif of the MaSp1 protein were amorphous. The results of FTIR analysis showed that the content of β-sheets in the silk of transgenic silkworms engineered with transgenic vectors with additional polyalanine was significantly higher than that of wild-type silkworm silk. Additionally, silk with a higher β-sheet content had better fracture strength and Young’s modulus. The mechanical properties of silk with longer chains of exogenous proteins were improved. In general, our results provide theoretical guidance and technical support for the large-scale production of excellent bionic silk.

## 1. Introduction

Silkworm (*Bombyx mori*) silk has been used in the textile industry for thousands of years, and it is the only natural silk fiber that can be produced on a large scale [1]. The silkworm silk is composed of fibroin and sericin. Fibroin is a hydrophobic protein that consists of a fibroin light (FL) chain (26 kDa), a fibroin heavy (FH) chain (350 kDa), and a glycoprotein called the P25 protein (30 kDa) [2,3,4]. It is generally believed that the mechanical properties of silk are mainly dependent on the FH chain in silk [5]. Experimentally, when the FH chain is destroyed, the mechanical performance of silk decreases sharply, supporting this hypothesis [6]. Molecular structure analysis shows that the FH chain has a large molecular weight and a large number of (GA)_N_GX repeat motifs, which can form a large crystal/semicrystal domain in the silk fiber and could be the molecular basis of the mechanical properties of the silk fibroin heavy chain [7,8].

Spiders produce different silks from seven silk glands to achieve biological functions such as stopping falls, catching prey, building webs, filling holes, and making eggshells/sacs [9,10]. The majority of studies analyzing spider silks have focused on the major ampullate dragline silks due to their high tensile strength and extensibility. The dragline silk fiber predominantly contains two types of conserved spidroins: ampullate dragline silk protein 1 (MaSp1) and major ampullate dragline silk protein 2 (MaSp2) [11,12]. More than 90% of the MaSp1 protein chain is a tandem repeat sequence consisting of four typical repeats of type 1, type 2, type 3, and type 4, which are formed by GGX and poly (A) motifs. The MaSp2 protein has a structure similar to that of the MaSp1 protein; it mainly consists of poly (A/GA) and GPGGX motifs [9,10,11,12]. Compared with the molecular structure of the FH chain, the main difference in the molecular structure of the spider dragline is the poly (A) domain with multiple repeats.

Bagworms are lepidopteran insects that use silk to build and hang their nests. As a material to bear the weight of the insect itself and the nest, bagworm silk shows a much higher strength and modulus than silkworm silk [13]. In addition to having a repeated motif similar to that of the silkworm fibroin heavy chain molecule (GA)_N_GX, the primary structure of bagworm fibroin also has the longest known poly (A) motif (22 residues) of any natural silk protein [14]. This long poly (A) structure aggregates the adjacent poly (A) helix together under hydrophobic interactions and self-assembles into a hexagonal arrangement, forming an ordered meridional periodic structure with the polyglycine motif [14]. This special structure induced by the long poly (A) could be the structural basis for the excellent mechanical properties of bagworm silk.

The high molecular weight and structural similarity of the FH chain with MaSp and the large-scale production of silk make silkworm an ideal bioreactor for spider silk protein. In recent years, through the *piggy*Bac transposon system, several small spider silk genes were randomly integrated into the silkworm genome and exhibited mechanical properties increase in the fibers [7,15,16]. Transcription activator-like effector nucleases (TALENs)/clustered regularly interspaced short palindromic repeats associated protein-9 nuclease (Cas9)-initiated homology-directed repair (HDR) have been established in *B. mori* to express recombinant spider silk protein, the extensibility and strength of the resulting silk fibers demonstrated significant improvements [3,6]. These advancements exhibit a promising means by which to produce spider silk protein. At present, people generally focus on introducing larger foreign gene fragments or improving the expression level of exogenous protein to further improve the performance of transgenic silk. However, technological bottlenecks will put a drag on the progress of these studies. Therefore, in order to meet the urgent commercial demand for high-performance silk materials, more research direction needs to be found.

Based on structural analysis of these natural fibers, the existence of a poly (A) structure may play a key role in the excellent mechanical properties of spider dragline silk and bagworm silk. This paper investigates if the long poly (A) motif in the fibroin molecules is, in fact, an important factor in determining the strength of silk fibers. To explore the molecular structure of foreign proteins that could improve the mechanical properties of silk fiber, the type 3 repetitive sequence of the *MaSp1* gene of *Latrodectus hesperus* was used as the basic structural unit, and several transgenic plasmids containing the fusion protein that consists of the FL light chain, type 3 repeats with different multiples and the poly (A) motif with different lengths were constructed. Three kinds of silk protein-based bionic spider silks were obtained by *piggy*Bac-mediated microinjection. The results show that bionic spider silk with a longer poly (A) motif has a higher mechanical strength. The experiment proves that poly (A) plays an important role in the stress properties of silk. The results will provide a new idea for research on the use of silkworms to express spider silk.

## 2. Results

### 2.1. Construction of the Transgenic Vector and Screening for Positive Silkworms

**Figure 1:**
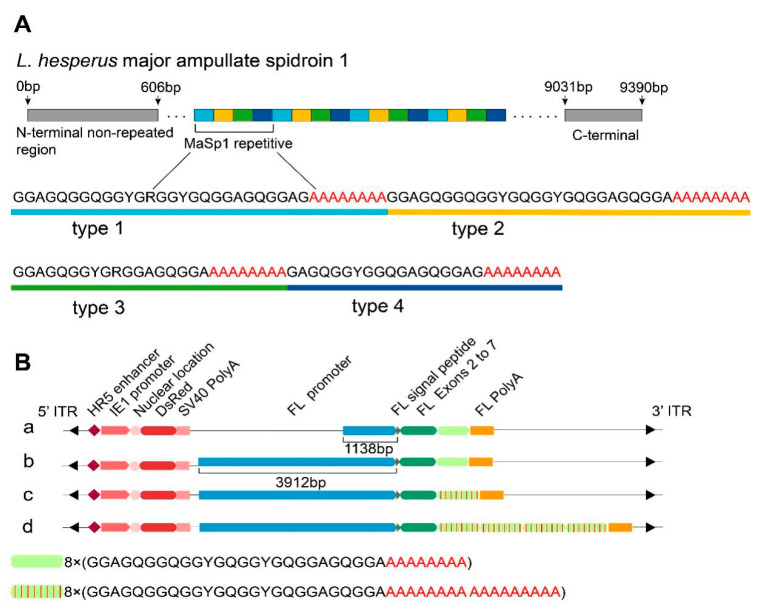
Schematic representation of the primary structure of *Latrodectus hesperus* major ampullate spidroin 1 and the expression cassettes in the four *piggy*Bac-derived transgenic vectors. (**A**) The primary structure scheme of *L. hesperus* major ampullate spidroin 1. (**B**) Schematic representation of the expression cassettes in the four *piggy*Bac-derived transgenic vectors. (**a**) The 8MT1 strain foreign gene expression cassette. (**b**) The 8MT2 strain foreign gene expression cassette. (**c**) The 8MTA strain foreign gene expression cassette. (**d**) The 32MTA strain foreign gene expression cassette.

The repetitive motifs of the MaSp1 protein from the black widow spider exhibit extreme sequence modularity [12]. These motifs can be divided into four highly conserved repetitive units known as type 1, type 2, type 3, and type 4 (Figure 1A). Each type is composed of a glycine-rich region followed by a poly (A) motif. We selected the type 3 module with a shorter overall length and a higher proportion of poly (A) as the basic unit and designed three transgenic vectors containing a 4 kb FL promoter to initiate exogenous gene expression (Figure 1B(b–d)). In addition, a vector containing an 8-fold type 3 gene and a 1 kb FL promoter was constructed (Figure 1B(a)).

The mechanical properties of silk are mainly contributed to the FH chain, but its high molecular weight (350 kDa) makes it unsuitable to be constructed as a fusion protein. In order to use the self-assembly behavior of silk fibroin to target foreign proteins into silk molecules, we fused the recombinant spider silk fibroin with the silk FL chain. The fusion proteins of the FL chain-8-fold type 3, FL chain-8-fold type 3-poly (A), and FL chain-32-fold type 3-poly (A) (Figure 1B(b–d)) had predicted MWs of 44.92 kDa, 50.03 kDa, and 112.36 kDa, respectively. The gene sequence of the multiple type 3 module was designed according to the codon preference of the FL chain. A red fluorescence protein gene (DsRed) activated by the IE1 promoter on the vector was used as the screening marker (Figure 1B).

**Figure 2:**
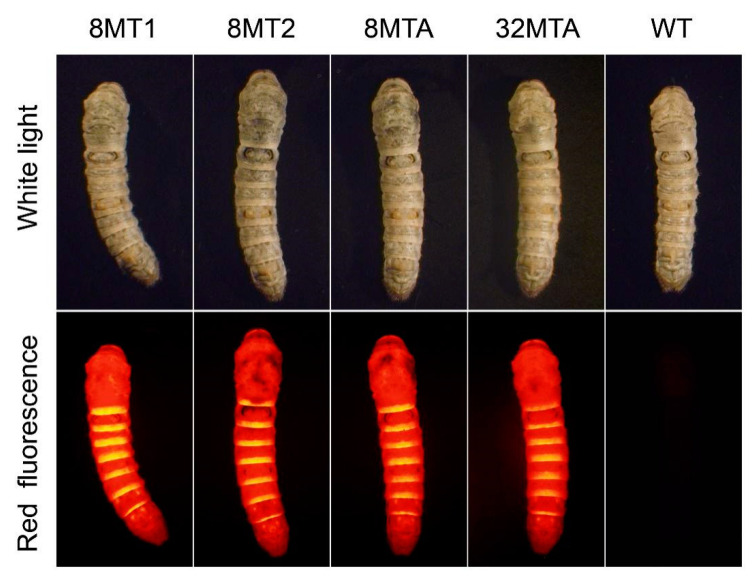
Positive transgenic silkworm strains with the red fluorescence protein gene (DsRed) protein expressed throughout their whole body. The third instar-positive silkworm strains and the WT strains (*Lan 10*) were viewed under white light and red fluorescence excitation wavelengths, respectively.

Each transgenic vector type, mixed with the helper plasmid, was microinjected into the fertilized eggs of *Bombyx mori*, and the positive silkworms corresponding to the vector was selected in the G1 generation by screening the systemic red fluorescence (Figure 2). The strains carrying the FL chain-8-fold type 3 with a 1 kb FL promoter were termed 8MT1. The strains carrying FL chain-8-fold type 3, FL chain-8-fold type 3-poly (A), and FL chain-32-fold type 3-poly (A) recombinant genes with the 4 kb FL promoter were termed 8MT2, 8MTA, and 32MTA, respectively. In each type of transgenic silkworm strain, the transgenic lineages with the brightest red fluorescence were selected and mated with wild-type (WT) moths to obtain the hybrid G2 generation. There were no significant differences between the cocoon shell weights among the four transgenic silkworm lineages within the G2 generation (Figure S1).

### 2.2. Analysis of Foreign Gene Insertion Sites in the Transgenic Silkworms

The vector insertion sites within the *B. mori* genome of the four silkworm strains were detected via inverse PCR. The results showed that all four transgenic lines had a single insertion site, and the integration sites were all located in the non-coding region of the silkworm genome (Figure S2). There was no significant phenotypic difference between the transgenic strains and the wild-type *Lan 10* throughout the whole life cycle.

### 2.3. Identification of Foreign Gene Expression in the Transgenic Silkworms

**Figure 3:**
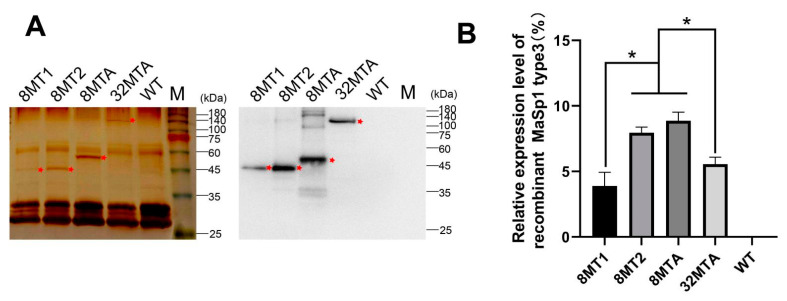
Expression identification and analysis of the recombinant spider silk protein in the silkworm cocoon shells. (**A**) Results of silver staining (left) and Western blot (right) of the degummed silkworm cocoon shells. M: 180 kDa protein Marker. (**B**) Expression analysis of the foreign protein relative to the FL protein through gray analysis using the Gel-Pro-analyzer4 software. The mean ± SD derived from three independent replicate experiments. The target protein band is marked by an asterisk. * represents *p* < 0.05 (Two-tailed unpaired *t*-tests).

To confirm that the recombined spider silk gene was expressed in the transgenic silkworm cocoon shells, the silks from 8MT1, 8MT2, 8MTA, and 32MTA were analyzed via silver staining and Western blotting. Protein bands with the predicted sizes were detected in the cocoon shells for all four strains, suggesting that the foreign proteins were successfully secreted into the cocoon shells (Figure 3A).

We calculated the relative expression level of the exogenous protein in the strains 8MT1, 8MT2, 8MTA, and 32MTA and used the silk fibroin light chain as the control (Figure 3B). Although the molecular weight of 8MT2 is similar to that of 8MT1, 8MT2 carries a 4 kb FL promoter, and its transcriptional activity is stronger than that of the 1 kb FL promoter. Hence, the expression of the 8MT2 strain is higher than that of the 8MT1 strain. The 8MT2 strain and the 8MTA strain carry the same size FL promoter, and the molecular weights of the foreign proteins were similar. Therefore, the expression level was also similar. Furthermore, the 32MTA strain carries the same 4 kb promoter as the 8MT2 and 8MTA strains, but the molecular weight of the foreign protein increased significantly, and the expression level decreased significantly.

### 2.4. Foreign Protein Structure Predictions

Many proteins or partial regions of proteins lack stable and well-defined three-dimensional structures under physiological conditions in vitro [17]. These proteins or regions are often called intrinsically disordered proteins (IDPs) or intrinsically disordered regions (IDRs). Due to the functional importance of intrinsic disorder proteins or protein regions, the study of these proteins is usually helpful to our understanding of protein function and the underlying mechanism. One efficient method to study the IDPs and IDRs is the use of disorder predictors. Hence, far, various researchers have built dozens of predictors. PONDR^®^ VL-XT is an early predictor, including three predictors [18]. Based on the database established by collecting the characterized ordered and disordered sequences, it can predict from the first residue to the last residue by the algorithm. PONDR^®^ VSL2 predictor is a more advanced prediction model, which is suitable for any length of the disordered region. As the 10-fold cross-validation results showed, the VSL2 predictors achieved well-balanced prediction accuracies of 81% on both short and long disordered regions [17,18].

**Figure 4:**
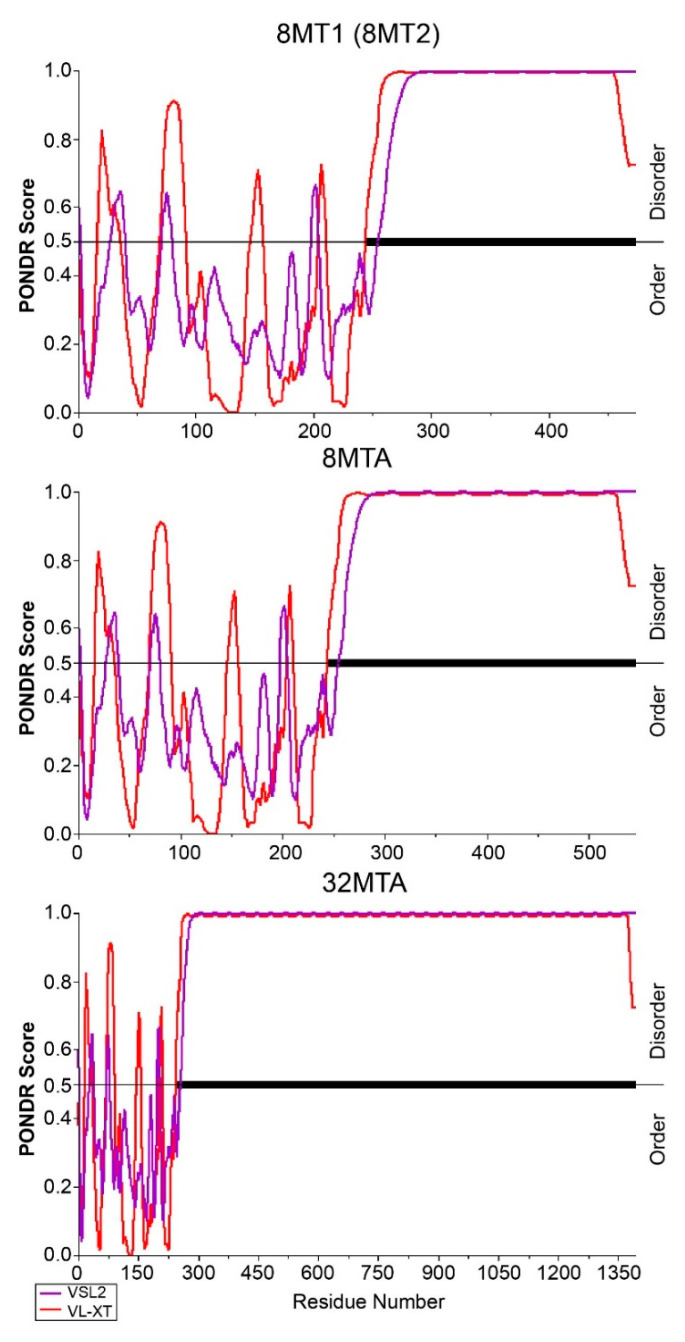
PONDR^®^ VSL2 and PONDR^®^ VL-XT prediction models for the four exogenous proteins. The regions with scores of 0.5 and above represent disorder. Residues 0–263 represent silk fibroin light chain protein, and the residues after 263 represent repeats of type 3 recombinant protein.

In this study, the protein disorder prediction tools PONDR^®^ VSL2 and PONDR^®^ VL-XT were used to predict the three types of exogenous proteins [17]. The results showed that the FL chain region in the fused protein showed alterations in order and disorder (Figure 4). However, the two predictors showed similar results in predicting that the Masp1 type 3 repeat regions of the three types of recombinant proteins were highly disordered and could not form a stable protein secondary structure (Figure 4).

### 2.5. Secondary Structure Analyses of the Transgenic Fibers

To characterize the molecular basis of the mechanical properties, the secondary structure content of each transgenic fiber type was investigated by FTIR. The infrared absorption spectrum for fibroin is mainly composed of the amide I band, the amide II band, and the amide III band, among which the amide I band at 1600–1700 cm^−1^ is the most valuable for investigating the secondary structure due to its high strength and resistance to being affected by the nature of the residual side chain [19,20]. To obtain a quantitative understanding of the compositions of different secondary structures, peak deconvolution analysis with the amide I vibration band was carried out. The peak parameters that be used in the deconvolution were established according to the literature [19,20,21,22]. Four Gaussian peaks were used to fit the broad amide I band, where two peaks for β-sheet structures locate at 1615–1645 cm^−1^ and 1690–1700 cm^−1^, one peak for helical/random coil structures located at 1645–1665 cm^−1^ and one peak for β-turn structures at 1680–1690 cm^−1^. After calculating the content of the secondary structure by means of peak-differentiating analysis, the quantitative analysis was carried out [21].

**Figure 5:**
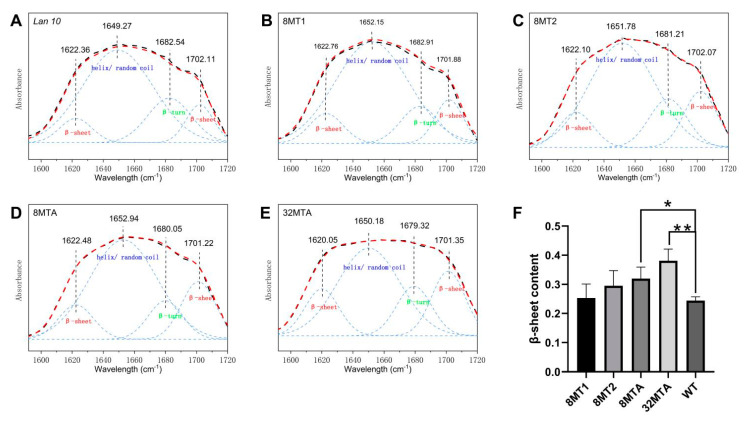
Secondary structural characteristics of the transgenic cocoon silks. (**A**) Deconvolution of the corresponding amide I band of the control. (**B**) Deconvolution of the corresponding amide I band of the 8MT1 strain. (**C**) Deconvolution of the corresponding amide I band of the 8MT2 strain. (**D**) Deconvolution of the corresponding amide I band of the 8MTA strain. (**E**) Deconvolution of the corresponding amide I band of the 32MTA strain. (**F**) β-sheet content of the transgenic cocoon silks. The mean ± SD derived from three independent replicate experiments. ** and * represent *p* < 0.01 and *p* < 0.05, respectively (Two-tailed unpaired *t*-tests).

As shown in Figure 5, the β-sheet content in the transgenic silk is more than the WT (*Lan 10*). For the transgenic silk fused with the same exogenous protein (8MT1 and 8MT2), then strain with a higher expression level of exogenous protein contained more β-sheet structures. For transgenic silkworm strains with similar expression levels of exogenous proteins (8MT2 and 8MTA), the strain with additional polyalanine domains showed a higher β-sheet content. However, the content of the β-turn structure showed the opposite trend (Figure 5). Furthermore, the transgenic silk strain 32MTA with the largest exogenous protein showed the highest β-sheet content.

### 2.6. Mechanical Properties of the Transgenic Fibers

The transgenic silk fibers were subjected to mechanical testing under identical controlled conditions, and their mechanical properties were evaluated relative to the control group (*Lan 10*) (Table 1 and Figure S3). The strengths of the four transgenic silks all were higher than that of the control group. For the three strains of silkworms (8MT1, 8MT2, and 8MTA) that were introduced with similar-sized exogenous genes, the 8MTA strain with an additional poly (A) structure had the best performance, with its maximum stress increased by 19.3% compared with the control group. The strength of the 8MT1 strain and the 8MT2 strain increased by 2.2% and 6.3% compared to that of the control group, respectively, showing a positive correlation between silk performance and exogenous protein expression. The 32MTA strain with the largest exogenous gene showed the most significant performance improvement, with the maximum stress increased by 32.9% compared to that of the control group, although its exogenous gene expression level was lower than that of the 8MT2 and 8MTA strains.

The fibroin elementary unit consists of six disulfide-linked FH and FL dimers and one P25 glycoprotein molecule. The FL dimer forms a disulfide bond between Cys172 in FL and Cys-c20 in FH and forms the fibroin unit on the endoplasmic reticulum [23,24]. We speculated that the FL fusion protein could form the elementary fibroin unit like nature FL chain (Figure S4). Therefore, we designed the fusion proteins of full-length FL chain and recombinant spider silk proteins and expected to provide a high-strength molecular basis for transgenic silk with the help of the characteristics of foreign protein. Notably, recently published papers have confirmed that the FL fusion protein can form the fibroin basic unit through non-covalent interactions with the NTD of Fib-H [24]. The results of this study confirmed that the strength of transgenic silk was significantly improved, which may be due to the above reason.

## 3. Discussion

In this study, we constructed vectors that contained a 4 kb FL promoter and a recombinant protein fused to the FL chain with the 8-fold natural *Latrodectus hesperus* Masp1 type 3 repeat, or the FL chain-8-fold type 3-poly (A), or the FL chain-32-fold type 3-poly (A). We generated transgenic silkworms and detected the target protein within the cocoon shells of the G2 generation transgenic silkworm strains to verify that the foreign gene had been successfully integrated into the silkworm genome and thus successfully inherited and expressed. Compared with the transgenic silkworms using the 1 kb FL chain promoter, the results showed that the expression levels of the foreign proteins within the transgenic silkworms 8MT2 and 8MTA increased by 2.04- and 2.28-fold, respectively. Even the transgenic silkworm 32MTA strain, which has an exogenous protein with a high molecular weight, reached an expression level 43% higher than the 8MT1, indicating that using long sequence promoters, such as the 4 kb promoter, is an effective method for improving the expression level of foreign proteins.

Disordered region predictors were used in analyzing the foreign proteins introduced in this study, and the results showed that the type 3 repeat sequence and poly (A) motif of the fusion proteins were amorphous regions that could not form a stable secondary structure. This is consistent with the conclusion of Joseph et al. [25] that the poly (A) region forms an amorphous polymer at room temperature and with the result of Jenkins et al. [26] that the proteins stored in the spider silk gland were arranged in a random coil based on analysis with high-resolution magic angle spinning NMR spectroscopy.

However, FTIR analysis showed that the β-sheet contents of the 8MTA and 32MTA strains are significantly higher than that of wild-type silkworm silk, and the β-sheet content in silk is directly proportional to the poly (A) content of the foreign protein. Therefore, we speculate that poly (A) repeats ultimately form β-sheet crystals within transgenic silk. Other studies on the molecular structure of natural silk containing long poly (A) repeats in the primary structure, such as spider silk, Bombycidae silk, and bagworm silk, also suggest that poly (A) forms β-sheet crystals in silk fiber [14,27]. The polyalanine module of foreign proteins in transgenic silkworms exists in an amorphous state within the silk gland and forms β-sheet structures in silk. One of the possible explanations for this is that the conformation of the silk protein changes in the process of silk-spinning due to dehydration, physical extrusion, shearing and the change in pH (from alkaline to acidic) [28,29,30,31,32]. The process is similar to the conversion of poly (A) to β-sheets in natural spider silk [14,33]. This result indicates that the fusion protein can complete a structural transformation similar to the natural spider silk molecules in the spinning process of the silkworm and demonstrates that in order to form high-strength silk fiber, the proteins must undergo a spinning process similar to that of the silkworm and spider.

We still need to consider other reasons for the difference between the results of PONDR and FTIR. The aggregation state of poly (A) changed sharply with different lengths and temperatures. At near-physiological temperature, A19 (19 alanine peptides) slowly grew into loose clusters, while A25 (25 alanine peptides) rapidly assembled into small stable oligomers. At high temperatures, A19 assembled into fibrils, but A25 precipitated as dense, micrometer-sized particles [25]. This sensitive transition characteristic of polyalanine peptide is difficult to predict accurately and may have an important impact on the properties and molecular structure of transgenic silk. Furthermore, fibroin easily forms an internal hydrophobic, externally hydrophilic micelle structure [24]. The surface of the micelles is negatively charged, which can prevent premature crystallization of silk in vivo. FL chain fusion protein may affect the formation of such micelles, resulting in premature crystallization of poly (A).

It is generally believed that β-sheet crystals contribute to the strength of the spider dragline silk. The poly (A) motif of the MaSp1 protein forms a β-sheet configuration, and the A residues are alternately distributed on both sides of the conformational skeleton. The hydrophobic interactions caused by this conformation closely links the poly (A) chains together, thus making the spider silk fibers have outstanding tensile strength [9,10,11,12]. This study showed that the content of β-sheets in the transgenic silk of the 8MTA strain was significantly higher than that of the wild-type, and the mechanical properties, such as the breaking strength and Young’s modulus, improved more in the 8MTA strain than in the 8MT1 strain, suggesting that the improvement in the mechanical properties of 8MTA silk is the result of increased β-sheet content.

The 8MT1 and 8MT2 showed a slight improvement in extensibility, while the 8MTA showed similar extensibility to the wild-type (Figure S3). Materials always tend to exhibit a balance between stiffness and extensibility, such as increasing stiffness at the expense of extensibility or vice versa [34]. Compared with 8MT1, 8MTA has higher stiffness (Young’s modulus), which may reduce its extensibility. The comprehensive improvement of mechanical properties in the 32MTA strain compared to the 8MTA strain, including strength, Young’s modulus, extensibility, and toughness (Table 1 and Figure S3), indicate that even with the same molecular structure, larger protein molecules will have better mechanical properties. Based on the research results, we can further clarify that, regardless of the molecular structure, the longer the molecule is, the better the mechanical properties are of the silk. This also explains why natural silk with excellent properties, such as strength and toughness, obtained by natural selection has very long molecular chains.

The stress–strain curves of 8MTA and 32MTA exist in the plateau region, which may cope with the structural transformation from α-helix to β-sheet (Figure S3) [35,36,37]. If this change is caused by the introduction of an additional poly (A) structure, it will be another interesting discovery. We will try to clarify this phenomenon in the following work.

Several studies have found that variability in alanine within a species of spider’s silk will not necessarily lead to protein structural changes or mechanical property variation. Moreover, alanine-containing structures can be reconfigured on the fly under subtle changes in spinning conditions [29,31]. The difference in this study is that we expect to introduce additional poly (A) motif into silk rather than increasing the amount of alanine. The properties and functions of protein are based on its stereostructure, which depends on the arrangement of amino acids. In this research, we introduce the polyalanine motif, which will determine the structure of the corresponding protein. Therefore, we think that the observed changes in the properties and structure of the silk fibers are related to the increase of poly (A). However, we are still not clear about the interaction mechanism between poly (A) and other motifs or the mechanism of rheological changes caused by polyalanine. Further study on the spinning process and silk structure of transgenic silkworm will provide more information on the molecular mechanism of strength provided by poly (A).

The different silk mechanical properties are related to the whole spinning process, including the pH value in the silk gland, the shear stress of silk fibroin flowing in the silk gland, the dehydration process of silk fibroin with the help of microvilli, the friction between silk and valve, etc. [29,31,32,38,39,40,41,42]. We fully considered these factors, so we ensured all experimental silkworm strains to be raised at the same time and strictly controlled the same feeding environment, food source and spinning environment. Thus, the influence of the environment on the properties of different varieties of silk is eliminated. After this, we think the experimental data are scientific.

This study confirmed the important role of the long poly (A) motif in enhancing the strength and modulus of silk fibers, which provided a new idea and direction for research on the use of silkworms to express spider silk and thus improve the performance of silk.

## 4. Materials and Methods

### 4.1. Vector Construction

The 3912 bp and 1138 bp primary promoters of the FL gene (pFL) were cloned from the genomic DNA of *B. mori P50* by PCR amplification. The two pairs of gene-specific primers (P-FL-4K (F/R) and P-FL-1K (F/R)) are shown in Table S1. The PCR products were inserted into the corresponding sites of the pBac-IE1-DsRed-SV40 transgenic vector and named pBac-IE1-DsRed-SV40-pFL-4K and pBac-IE1-DsRed-SV40-pFL-1K. The transgenic vector pBac-IE1-DsRed-SV40 is a *piggy*Bac-driven plasmid preserved in our laboratory.

The DNA fragments FL exons-8×masp1 type 3-FL poly (A) and FL exons-8× (masp1 type 3+9A)-FL poly (A) were synthesized (GENEWIZ, Suzhou, China), and the FL exons-32× (masp1 type 3+9A)-FL poly (A) was obtained by a doubling strategy [43]. Then, the fragment of FL exons-8×masp1 type 3-FL poly (A) was inserted into pBac-IE1-DsRed-SV40-pFL-4K and pBac-IE1-DsRed-SV40-pFL-1K. The fragment of FL exon-8×(masp1 type 3+9A)-FL poly (A) and FL exon-32×(masp1 type 3+9A)-FL poly (A) was then inserted into pBac-IE1-DsRed-SV40-pFL-4K. Finally, we obtained four *piggy*Bac-driven transgenic vectors: pBac-IE1-DsRed-SV40-pFL-4K-8-type 3, pBac-IE1-DsRed-SV40- pFL-4K-8-type 3-9A, pBac-IE1-DsRed-SV40-pFL-4K-32-type 3-9A, and pBac-IE1-DsRed-SV40-pFL-1K-8-type 3. Each vector was identified by sequencing (GENEWIZ, Suzhou, China), and all DNA manipulations were carried out according to standard protocols.

### 4.2. Silkworm Transformation and Identification

*B. mori Lan 10* insects were used for transgenic experiments according to a previously described method [44,45]. The preparation of the transgenic plasmid and the helper plasmid was also performed according to our previously described method [7]. The piggyBac-derived transgenic vector and the helper vector were mixed at a proportion of 1:1.5 and microinjected into generation 0 (G0) embryos of *Lan 10* within 2 h after oviposition. After the microinjection experiment, the embryos were incubated at 25 °C for 7–10 days. The larvae were reared with fresh mulberry leaves at 25 °C and 80% humidity.

The G0 transgenic moths were mated with WT moths, and the positive transgenic silkworms were screened in the G1 larval stage through fluorescent gene expression (IE1-DsRed) by fluorescence microscopy (Olympus SZX16, Tokyo, Japan). The positive G1 larvae were fed fresh mulberry leaves at 25 °C and 80% humidity and then hybridized with the wild-type at the adult stage. At the fifth instar of G2, the positive silkworm lines corresponding to each transgenic vector with the brightest fluorescence were selected for this study.

### 4.3. Western Blotting

Soluble silk fibroin from the cocoon shells was prepared by modifying a previous method [7]. Briefly, the cocoons were degummed in a 0.05% (wt/vol) Na_2_CO_3_ solution, and the degummed silk was dissolved in a 9.3 M aqueous lithium thiocyanate containing 2% (vol/vol) β-mercaptoethanol at a ratio of 50:500. After incubation, the samples were dialyzed with deionized water overnight. The obtained sample was then centrifuged, and the supernatant was used for the next experiment.

A sample containing 50 µg of the total protein was mixed with an electrophoresis buffer at a ratio of 1:1 and incubated at 50 °C for 10 min. The samples were separated by a 4–15% gradient SDS–PAGE. After separation, the proteins were blocked onto PVDF membranes using a GE transfer cell according to the manufacturer’s instructions. The PVDF membrane was blocked with 5% nonfat milk powder dissolved in TBS-T (10 mM Tris, 150 mM NaCl, and 0.1% Tween-20). Then, the specific polyclonal antibody of the MaSp1 type 3 protein (diluted at 1:5000) was used as the primary antibody (GenScript, Nanjing, China), and peroxidase-conjugated goat anti-rabbit IgG-HRP (1:8000 dilution) was used as the secondary antibody (GenScript, Nanjing, China). The primary antibody was specific to the “CGAGQGGYGRGGAGQ” sequence corresponding to the type 3 protein. The ECL luminescence reagent (Sangon Biotech, Shanghai, China) was used for signal detection.

To further identify the content of the recombinant spider silk protein in the transgenic silk fibers, we quantified the relative amount and estimated the ratio of the targeted protein against the fib-L protein as described previously [16]. Three independent repeats were performed on all the samples.

### 4.4. Prediction of the Protein Disordered Regions and FTIR Spectroscopy Analysis

The disorder degree of the exogenous protein was predicted on the website http://www.pondr.com/ (accessed on 2 February 2021). The preparation of the degummed silk material is described in this paper. A total of 2 mg dried degumming silk was mixed with 200 mg of potassium bromide (KBr), ground thoroughly in a mortar, and then dried. The mixture was pressed into tablets for testing. The FTIR spectra were recorded using a Fourier-transform infrared spectrometer (FTIR-8400S, Shimadzu, Tokyo, Japan). The measurements were performed with a wavenumber ranging between 400 and 4000 cm^–1^, a resolution of 4 cm^−1^, and 32 scans. Spectral smoothing and peak deconvolution analysis was performed using the OMNIC 8.2 software.

### 4.5. Mechanical Properties Testing of the Transgenic Silk

Ten medium-sized cocoons were selected from each transgenic strain, and five silk fibers were taken from each cocoon for mechanical testing. Single fibers were obtained in accordance with a previous description [46]. Briefly, the cocoons were immersed in 100 °C deionized water for 2 min and then immersed in 65 °C deionized water for 3 min. Subsequently, the cocoons were immersed in 100 °C deionized water for another 2 min and finally cooled in 85 °C deionized water for 15 min. The changes in water temperature softened the sericin and allowed water to fill the space between the silk fibers. Preparation before tensile testing was performed as previously described [47]. A single silk fiber was attached to a rectangular frame with a distance of 30 mm between the two ends of the fiber to ensure an initial length of 30 mm. The cross-sectional diameter of each silk sample was measured with a digital microscope (VHX-600, Keyence, Osaka, Japan) at 1000× magnification. To ensure the accuracy of measurement, each sample was measured five times, and the average value was used to calculate the cross-sectional area of each sample; this area measurement was then used as a parameter to calculate the mechanical properties of the silk sample.

The AGS-J Universal Test instrument (Shimadzu, Tokyo, Japan) was used, and a 5 N load cell was equipped for tensile testing with a constant rate of 2 mm/min and a frequency of 250 MHz. Before the test, the rectangular frame was fixed with the silk vertically between the upper and lower clamps of the test instrument. Next, the two long sides of the rectangular paper frame were carefully cut off so that the tension could be transferred to the silk fiber. The test was performed at room temperature, and the experimental data were automatically recorded by the software (Trapezium2, Shimadzu, Tokyo, Japan). The maximum stress, maximum strain, and Young’s modulus were calculated by Trapezium2. The toughness was calculated by the area integral under the stress–strain curve (Origin2019b).

### 4.6. Statistical Analyses

GraphPad Prism 7 and SPSS 23 were used for statistical analysis. Two-tailed unpaired t-tests were used to compare data sets.

## 5. Conclusions

The key conclusions indicated in this study are as follows: (1) The polyalanine motif of the foreign protein exists in the silk gland of *Bombyx mori* in an amorphous state, which is transformed into a β-sheet structure during silk-spinning; (2) Integration of the Latrodectus hesperus MaSp1 gene repeats into the silkworm genome, especially those with an additional polyalanine, can significantly increase the content of β-sheets and further improve the strength and Young’s modulus of transgenic silk; and (3) When an amino acid motif has the function of improving the mechanical properties of silk fibers, the longer and more repetitive motifs result in better silk performance, whether natural or “engineered”.

## Figures and Tables

**Table 1:** Average mechanical properties of transgenic silk fibers.

Silkworm Strains	Max Stress (MPa)	Young’s Modulus (MPa)
WT	179.62 ± 25.78	3283.97 ± 1045.32
8MT1	183.56 ± 19.59	3379.44 ± 783.01
8MT2	190.95 ± 15.04	3538.23 ± 503.71
8MTA	214.02 ± 25.03	4321.43 ± 1380.56
32MTA	238.72 ± 13.98	4631.16 ± 611.72

## Data Availability

Data are contained within the article or Supplementary Material The data presented in this study are available in the insert article or Supplementary Material here.

## Supplementary Materials

The following are available online at https://www.mdpi.com/1422-0067/22/4/1513/s1, Figure S1: Cocoon shells weight of transgenic silkworm lineages and WT, Figure S2: Genomic insertion sites of the four transgenic lineages, Figure S3: Stress−strain curves of silk fibers, Figure S4: Model of natural silk elementary unit and transgenic silk elementary unit with fusion protein, Table S1: The primers used in this study.
