# Supplementary material for: Recombinant Silk Proteins with Additional Polyalanine Have Excellent Mechanical Properties
Source: Int J Mol Sci. 2021 Feb 3;22(4):1513. doi: 10.3390/ijms22041513 (PMC7913374; doi:10.3390/ijms22041513)
Supplement: Supplementary file 1 [file ijms-22-01513-s001.pdf]

International Journal of
Molecular Sciences

MDPI

Recombinant silk proteins with additional polyalanine have excellent mechanical properties

Shuo Zhao[1], Xiaogang Ye[1], Meiyu Wu[1], Jinghua Ruan[1], Xiaoxiao Wang[1], Xiaoli Tang[1] and Boxiong Zhong[1,*]

1 College of Animal Sciences, Zhejiang University, Hangzhou310058, P. R. China; asd8899176@163.com (S.Z.) ; 11817001@zju.edu.cn (X.Y.) ; 11617008@zju.edu.cn (M.W.); jinghuaryan@icloud.com (J.R.); 21717412@zju.edu.cn (X.W.); xltang2017@163.com (X.T.); bxzhong@zju.edu.cn (B.Z.)

* Correspondence: bxzhong@zju.edu.cn; Tel.: +86-571-86971302

This file includes:

Supporting Information

Original Images for Blots and Gels

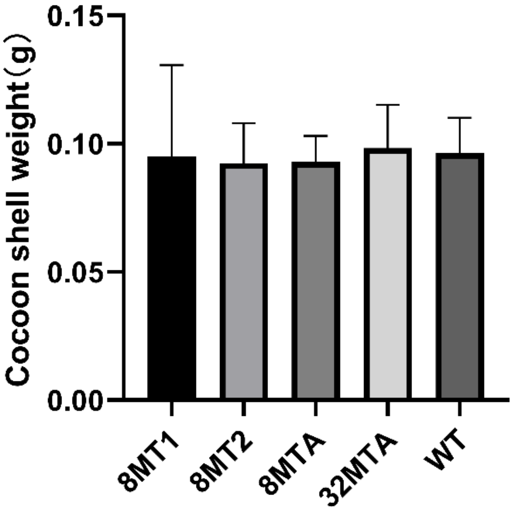

Figure S1. Cocoon shell weight of transgenic silkworm lineages and WT.

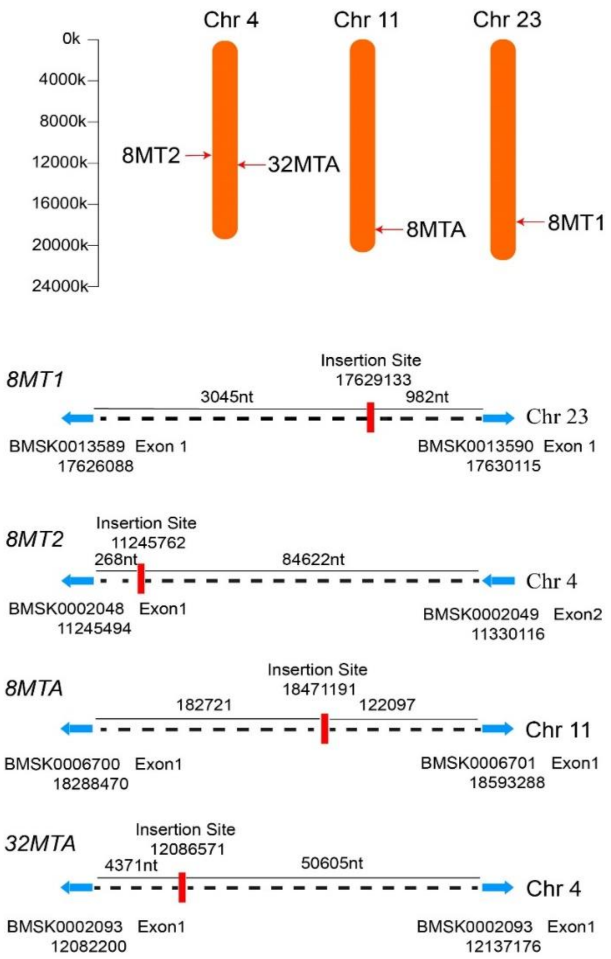

Figure S2. Genomic insertion sites of the four transgenic lineages.

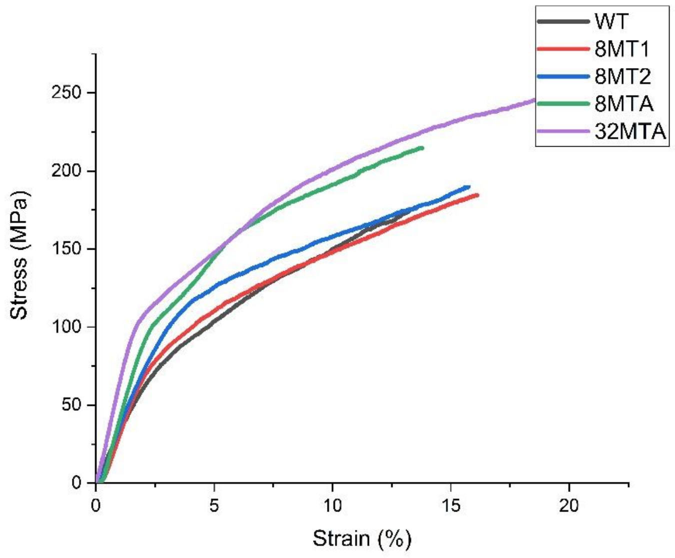

Figure S3. Stress–strain curves of WT and transgenic silk fibers.

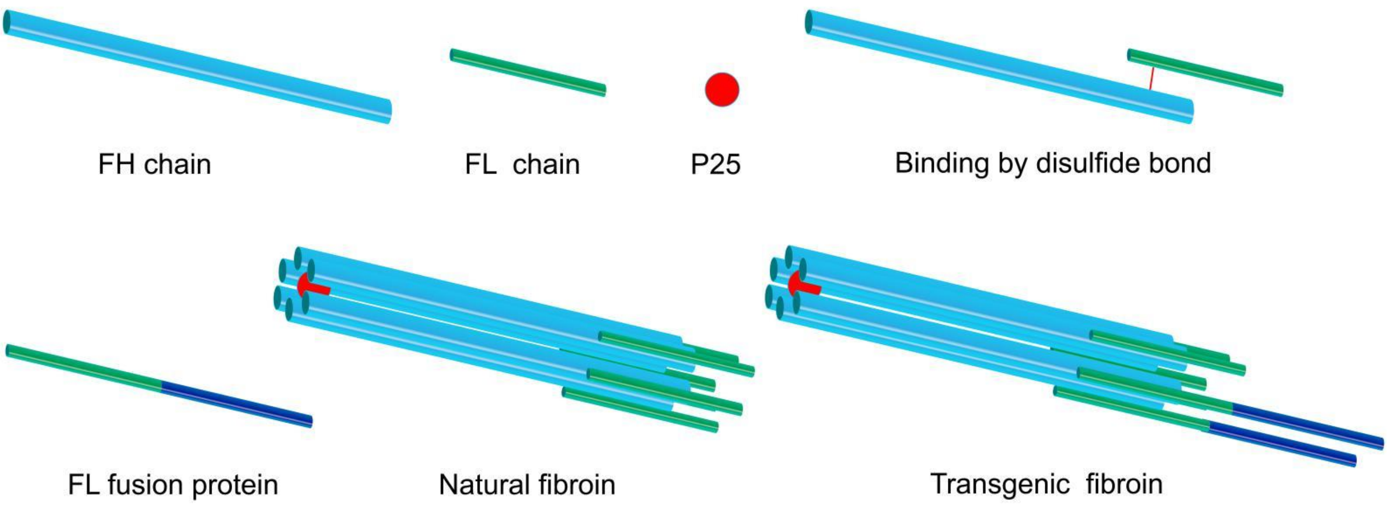

Figure S4. Model of natural silk elementary unit and transgenic silk elementary unit with fusion protein.

Table S1. The primers used in this study

Primer name	Sequence (5'-3')
P-FL-4K-F	TTAATTAAGAATCCAAGTTAGTTCAAGGTCC
P-FL-4K-R	ACTAGTTTTAGTGGTCTGTTATGTGACC
P-FL-1K-F	TTAATTAAGAGAAAATGCTCCACGTGAGTT
P-FL-1K-R	ACTAGTTTTAGTGGTCTGTTATGTGACC
L-reverse-1-F	GACAAGCACGCCTCAGCC
L-reverse-1-R	TGAGTCAAAATGACGCATGATTATC
R-reverse-1-F	TCTGTATATCGAGGTTTATTTA
R-reverse-1-R	CCGATAAAAACACATGC
L-reverse-2-F	GCTCCAAGCGGCGACTG
L-reverse-2-R	GGGATGTTCTTTAGACGATGAGC
R-reverse-2-F	ACTCAAAATTTCTTCTAAAGTAACAA
R-reverse-2-R	CTTTAACGTACGTCACAATATG

Original images for blots and gels

The unprocessed original images for blots and gels of Figure 3

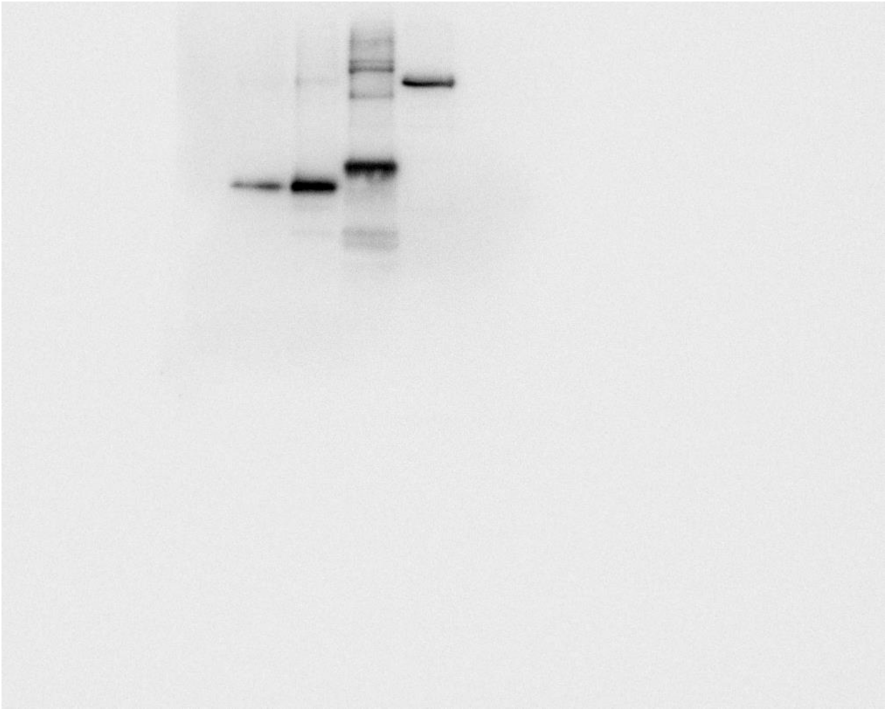

The original images for blots of Figure 3

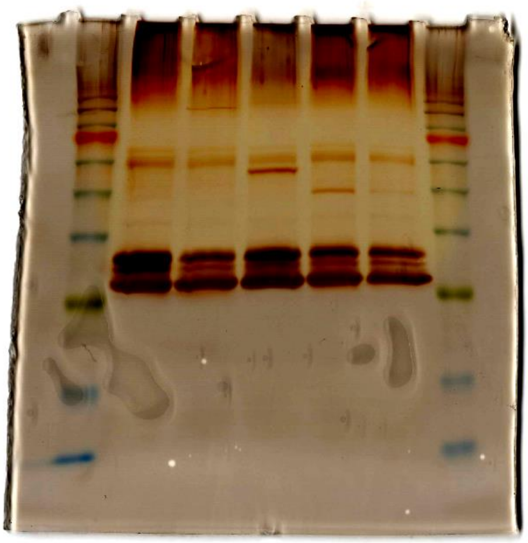

The original images for silver staining of Figure 3
